# Impact of financial development and institutional quality on remittance-growth nexus: evidence from the topmost remittance-earning economies

**DOI:** 10.1016/j.heliyon.2022.e11860

**Published:** 2022-11-29

**Authors:** Md. Saiful Islam, Ibrahim A. Alhamad

**Affiliations:** a Department of Economics and Finance, College of Business Administration, University of Hail, P.O. Box 55476, Hail, Saudi Arabia; b Management and Information Systems Department, College of Business Administration, University of Hail, Saudi Arabia

**Keywords:** Personal remittance, Financial development, Institutional quality, Economic growth

## Abstract

This study investigates the asymmetric impact of personal remittances on economic growth (EG) having financial development (FD) and institutional quality (INQ) as control variables using panel data from 1996 to 2019 on the ten largest remittance-earning economies of the world. It employs the pooled mean group (PMG) technique and Dumitrescu-Hurlin (D-H) causality check. The outcomes reveal that personal remittances asymmetrically impact economic expansion, the positive shocks of remittances cause EG negatively, while its negative shocks affect the latter positively with an overall positive impact. Both FD and INQ influence the long-run EG positively. No evidence of the threshold effect of FD on economic expansion is found. The D-H causality test produces several bidirectional and unidirectional causalities. Remittances and INQ directly cause EG, while FD causes it indirectly through the INQ channel. The outcomes offer implications for policymakers to continue developing their “financial markets and institutions” to improve the “access, depth, and efficiency” of financial services, progress INQ, and strive for more remittances to augment EG.

## 1 Introduction

Remittance refers to any transfer of money/income by someone employed abroad to his home country. It constitutes one of the leading sources of foreign income for the people of developing nations, frequently surpassing any other source of foreign finance. Relative to any other source of foreign finance, remittance is risk-free and involves no reimbursement. Therefore, remittance inflows to home countries create an essential source of finance for huge developmental economic activities in developing countries. Personal remittances generate many constructive spillover effects such as reducing poverty and promoting economic growth (EG), through augmenting disposable income and consumption of the remittance recipient households.

The global remittance amounted to 702 billion US dollars in 2020, in which the ten largest remittance-receiving countries namely, India, China, Mexico, Philippines, Egypt, Pakistan, France, Bangladesh, Germany, and Nigeria received a net remittance inflow of 83.1, 59.5, 42.9, 34.9, 29.6, 26.1, 24.5, 21.8, 17.9 and 17.2 billion US dollars respectively totaling 357.5 billion, and 50.93% of the world's total remittances. The volume of inward personal remittances is contingent on the migrants' capability, and their inspiration to remit money back to their home countries. Their motivation for remittance is related to their tie with families and the sizes of families. Other motives include philanthropic activities and self-interest including personal saving goals, and portfolio matters (OECD, 2006). Despite personal remittance's significant contribution to foreign income, which meets the much-needed funds for development efforts, its influence on economic expansion is not well explored. This study strives to examine the EG and personal remittance nexus in the presence of financial development (FD) and institutional quality (INQ) as control variables.

The influence of remittance inflows on FD is well-researched, however, the influence of FD on personal remittances is rarely studied. Generally, personal remittances are transferred through two channels, e.g., official/formal, and unofficial/informal channels. Remittances transferred through an official channel such as the banking sector usually contribute to FD, while remittances routed through the informal channels usually do not come across the financial system and hence, do not influence the FD. By contrast, an innovative financial system very often attracts idle surplus resources to innovative financial products. Moreover, an efficient and reliable financial system always fascinates the migrants to remit their money through the official channels and helps to curtail the use of informal avenues of remittance transfer. Remittances irrespective of the transmission route, impact EG either positively or negatively, based on their proper uses. Thus, a well-developed financial system is capable enough to disseminate proper information regarding financial products and promising investment opportunities and attract both formally and informally routed remittances and facilitate the desired allocation of financial resources, and spurring EG (Deodat, 2011; Sufian and Moise, 2010). Thus, FD has a significant role to uphold the remittance-growth relationship. Therefore, this paper particularly targets to explore the influence of FD in the remittance-growth nexus.

The influence of institutions on an economy's economic expansion was elevated first by North (1991). He drafted a thought-provoking discussion on the role of institutions in economic progress. Institutions refer to constraints shaped by a society that generates various socioeconomic, and political interfaces, and comprise diverse official and casual rules and bans (North, 1991). The formal and administrative structure in which persons, families, companies, and government efforts jointly to produce output, earnings, and proceeds and determine economic growth demarcates the institutional framework. Following North, the importance of INQ for EG of any country has appeared as a significant matter of interest.

OECD (2001) revealed that the quality of institutions shapes countries’ economic events; it involves law, human rights, governance quality, and services. This, in the long-term, INQ and EG maintain each other. Empirical evidence demonstrates that countries with good institutions stay more effective in realizing economic expansion. Iheonu et al. (2017) emphasized the requirement of strong institutions to achieve sustainable EG. However, there is a substantial dearth of proof to investigate the influence of INQ on EG. Hence, this study aims to scrutinize the impact of INQ on the remittance-growth nexus.

**Figure 1:**
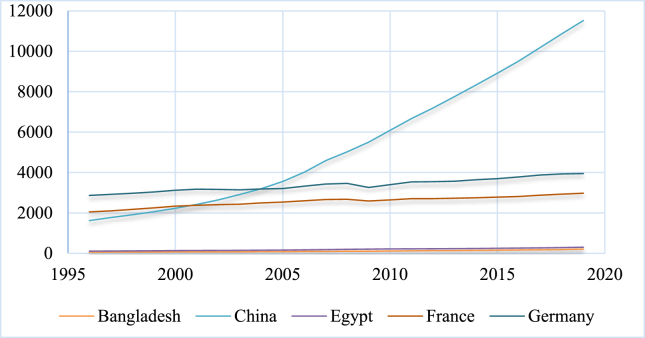
GDP in 2010 billion US dollars.

**Figure 2:**
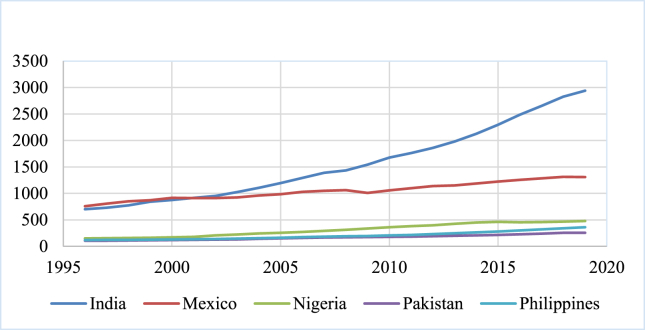
GDP in 2010 billion US dollars.

**Figure 3:**
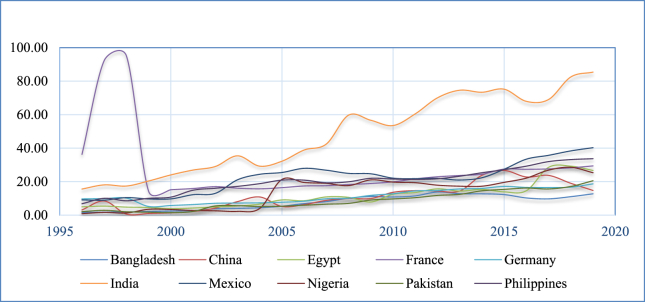
Remittances in 2010 billion US dollars.

The following figures [Figures 1, 2, and 3] highlight the volume of GDP and remittances in 2010 billion US dollars in the highest ten remittance-receiving countries. The top ten remittance-receiving nations include India, China, Mexico, Philippines, Egypt, Pakistan, France, Bangladesh, Germany, and Nigeria respectively in 2020 (World Bank, 2021). Both the GDP and remittances show almost rising and similar trends across the countries, indicating an apparent positive nexus between them. The current study is focused to identify this nexus in the presence of FD and INQ.

The subsequent parts of the paper are prepared in the following way. Part 2 assesses the pertinent literature, part 3 outlines the data and methods, part 4 discusses empirical outcomes, and eventually, part 5 finalizes the study with a conclusion.

## 2 Literature review

In this section, the literature on FD-growth, INQ-growth, and remittance-growth are examined in sequence.

### 2.1 FD and economic growth

Many studies have explored the nexus between FD and EG. Arestis et al. (2001) used time-series quarterly data on France, Germany, Japan, the UK, and the USA, employed the vector auto-regression (VAR) method, and examined the association between stock market progress and economic expansion, having banking system and stock market volatility as control variables. They revealed that while both the banks and stock markets were able to stimulate economic expansion, the impact of the stock market was less powerful. Estrada et al. (2010) used panel data on 125 developing Asian countries, applied the total factor production regression method, and examined the effect of FD on EG in Asia. They revealed that FD in terms of a deeper and diversified financial system exerted a significant positive influence on EG. Masoud and Hardaker (2012) employed panel data from 1995 to 2006 on 42 emerging countries and investigated FD and economic expansion relationships. The study revealed that stock market progress significantly enhanced economic expansion, and there was a long-term affiliation between stock market progress and economic development.

Samargandi et al. (2015) utilized panel data on 52 middle-income economies, employed the pooled mean group (PMG) estimation, examined the dynamic association between “FD and economic expansion” and reported a long-term reversed U-shaped association between finance and economic expansion, suggesting excessive finance could be detrimental to growth. Qamruzzaman and Jianguo (2017) employed yearly data from 1980 to 2016, utilized the ADRL bounds test and Granger causality, and examined the contribution of financial innovation to EG in Bangladesh. They used conventional proxies such as bank credit to the private sector and broad to narrow money as a share of GDP for financial innovation, and exhibited their positive contributions to EG, and also feedback relationship between the two proxies and EG. In a similar study, Puatwoe and Piabuo (2017) employed three proxies, e.g., domestic credit to the business sector, broad money, and deposit-GDP ratio for FD, using the ARDL technique, and examined their impacts on EG in Cameroon using annual data from 1908 to 2014. They revealed the long-run positive contribution of all three indicators of FD to EG.

Peprah et al. (2019) utilized yearly data from 1984 to 2015, applied the ARDL technique, and surveyed the relationship among FD, personal remittances, and economic expansion in Ghana. They revealed that the impact of remittance on EG was positive, while the impact of FD was significant in the short term and insignificant in the long term. However, the joint effect of remittance and FD was much more growth-enhancing, while excessive financial booms were detrimental to EG. Olayungbo and Quadri (2019) employed panel data from 2000 to 2015 on 20 Sub-Saharan African (SSA) states and explored the relationship among personal remittances, FD, and EG. The study utilized the PMG estimation and VAR-based Granger causality test, but in the presence of panel dependency, it failed to employ any second-generation unit root test. It revealed a positive contribution of remittances to EG, one-way causation from “FD to EG”, and no causality between remittance and FD. The study also revealed a negative impact of interaction term [remittance & FD] both in the short and long runs. Misati et al. (2019) used quarterly data from 2006 to 2016, applied the ARDL approach, studied the influence of personal remittance on FD in Kenya, and demonstrated a contributory impact of remittances on the latter.

Matei (2020) employed panel data from 1995 to 2016 on 11 emerging European economies, used the PMG model, and investigated FD and growth relationships. The study revealed that for linear modeling, FD impacts EG positively only in the short term, while for a non-linear setup, it generated a reversed U-shaped relationship with economic expansion. Rahman et al. (2020) employed the Markov Switching model and examined the influence of FD on economic expansion in Pakistan using yearly data from 1980 to 2017. The outcomes revealed that the influence of FD on growth was positive and more profound in high-growth regimes than in low-growth regimes. Abeka et al. (2021) utilized panel data on 44 SSA states from 1996 to 2017, applied the system GMM technique, and examined the “FD and EG” relationship. They revealed that FD empowered by telecommunication infrastructure positively affected the EG of SSA economies.

### 2.2 INQ and economic growth

The role of institutions on EG was first time emphasized by North (1991), who defined institutions as me-made constraints, which shape socioeconomic and civil interactions and are comprised of official rules and informal controls. He sketched the thought-provoking role of institutions and EG and change. Since then many studies followed him to examine the influence of INQ on EG.

Alexiou et al. (2014) employed Sudanese yearly data for the period 1972–2008, applied the political freedom index to measure institutional quality, utilized the ARDL bounds experiment, and investigated the relationship between INQ and EG. They demonstrated that the quality of institutions endured was determining factor in economic performance in Sudan. Nawaz et al. (2014) utilized data from 1996 to 2012 for a panel of 35 Asian countries, applied the GMM method, and studied the effect of the quality of institutions on economic expansion. They documented a substantial impact of institutions on the latter that varied across countries according to their stage of economic growth. Nguyen et al. (2018) also applied panel data concerning 29 emerging economies from 2002 to 2015, employed the system GMM technique, and discovered a noteworthy positive effect of INQ on economic expansion. However, they further reported that INQ foiled the positive influence of FDI and trade on economic expansion, and therefore, suggested improvement of INQ.

Salman et al. (2019) used panel data from 1990 to 2016, for three East Asian countries, investigated the effect of quality institutions on the growth-emissions nexus, and highlighted that INQ enriched EG. They also reported one-way causation from the quality of institutions to economic growth. Marija (2020) scrutinized the effect of INQ on economic expansion in EU and non-EU economies of southeast Europe and compared them. The study proxied the “worldwide governance indicators” for the quality of the institution, over the period 1996–2017, and employed the panel ARDL procedure. The outcomes revealed that in both the EU and non-EU economies INQ mattered EG.

Abubakar (2020) utilized time-series data over the period 1979–2018, applied the OLS method, scrutinized the consequence of “INQ on EG” in Nigeria, and conveyed that INQ stimulated economic progress. Zhao et al. (2021) observed the impact of formal reforms on developing countries’ investment and economic expansion. They applied panel data from 1996 to 2019 on 122 developing countries, considered economic and political reforms, and demonstrated that economic reforms were prudential for economic growth, while political reforms remained ineffective to ease economic crises. In a recent study, Islam and Shindaini (2022) used an ARDL framework, examined the effects of INQ and human capital on EG in Bangladesh, and found that INQ stimulated the long-run EG.

### 2.3 Remittance and economic growth

Many studies have examined the “remittance-EG nexus” in a panel setup, while others considered country-specific studies based on time series data. Several studies reported personal remittances’ positive contribution to EG. For example, Pradhan et al. (2008) considered a panel of 39 developing economies, used data from 1980 to 2004, applied fixed effects and random effects models, explored the impact of personal remittances on economic expansion, and determined that remittances exerted a positive impact on EG. Giuliano and Ruiz-Arranz (2009) utilized panel data on 100 developing economies from 1975 to 2002, used the system GMM, examined the affiliation among economic expansion, personal remittances, and FD, and determined that remittances promoted economic expansion in financially less advanced countries.

Fayissa and Nsiah (2010) employed panel data on 36 African nations from 1980 to 2004 and explored the impact of personal remittances relative to foreign capital on economic expansion. They showed that personal remittances positively impacted EG; a 10% rise in remittance inflow, contributed to a 0.4% growth in per capita income. Cooray (2012) conducted a panel study on six South Asian countries using annual data from 1970 to 2008, and an enhanced neoclassical growth model. The study explored the effect of remittances on economic expansion and demonstrated that remittances remarkably improved economic progress through the expansion of teaching-learning and financial development. In a country-specific study, Islam et al. (2012) carried out a primary investigation and surveyed the effect of personal remittances on the pastoral economy of Bangladesh. The study exhibited a positive influence of remittances in the rural economy of Bangladesh as it enhanced household consumption, and funded children's schooling and household health expenditures. In a similar study, Islam and Imran (2013) utilized annual data from 1971 to 2010 and studied the relationship between personal remittance, and per capita income in Bangladesh. They conducted a cointegration check and causality test and reported a feedback relationship between personal remittance and per capita income.

In a similar panel study on 24 Asia and Pacific countries, Imai et al. (2014) employed longitudinal information from 1980 to 2009, utilized the system GMM procedure, and tested the effect of personal remittance on economic expansion. They conveyed that remittances directly improved the poverty situation and enhanced EG, while the instability in remittance inflows harmed EG. Azam (2015) scrutinized the influence of personal remittance inflows on economic expansion in selected South Asian economies, i.e., Bangladesh, Sri Lanka, Pakistan, and India using yearly data from 1976 to 2012. The study employed the OLS procedure, with no further causality check, and demonstrated a positive impact of personal remittances on economic expansion. Kruah (2017) studied the association between economic expansion and personal remittance in Liberia utilizing monthly data from 2009 (01) to 2016 (06), employed the VAR model, and revealed personal remittance's positive contribution to the economic expansion of Liberia.

Chowdhury (2016) considered a panel of 33 highest remittance-earning developing economies, used data for the period 1979 to 2011, investigated the remittance-growth nexus, and determined a positive affiliation between economic expansion and remittance inflows. Adnan et al. (2020) considered a panel of 58 low, lower-middle, and middle-income nations, used data from 1988 to 2014, applied the GMM technique, and examined the remittances-growth nexus using FD and governance as control variables. The study revealed that remittance contributed to economic expansion positively in all income clusters of countries. Ekanayake and Moslares (2020) utilized a panel of 21 Latin American nations, used data from 1980 to 2018, applied the panel least square (PLS) and fully modified OLS methods, and observed the influence of remittance inflows on income expansion, and poverty. They revealed that workers’ remittance earnings accelerated economic development in the long run in most nations.

In a recent study, Chuc et al. (2021) investigated the combined effect of remittance arrivals and inclusive finance on the economic expansion of 60 low and middle-income economies employing annual data from 1996 to 2017. They employed the PLS method and fixed effects estimation and revealed that the growth-augmenting influence of remittance inflows was reinforced if financial inclusion was ensured. Jushi et al. (2021) examined the contribution of personal remittances to economic expansion in selected Western Balkan economies, i.e., Albania, Bosnia and Herzegovina, Croatia, Greece, Kosovo, Macedonia, Montenegro, and Serbia. They utilized data from 2000 to 2017, employed the VAR technique, and exhibited personal remittances’ positive influence on economic expansion. Islam (2021) investigated the remittance-growth nexus in four South Asian countries employing a panel framework for data from 1986 to 2019. The study employed a panel generalized least square method and Dumitrescu-Hurlin (D-H) causality check among others and exhibited positive support from remittances to economic expansion.

Thus, plenty of studies highlighted the positive contribution of personal remittances to economic progress. A few of them, however, highlighted either negative or no impact of remittance on economic expansion. For example, Chami et al. (2003) employed panel data on 113 countries during 1970–1998, explored the effect of remittances on economic activities, and concluded that migrants’ remittances negatively impacted the growth in per capita income. The study highlighted three stylized facts: “a significant proportion of remittances was spent on consumption; a smaller portion of remittances was used as saving or investment, and savings and investments out of remittances were used in unproductive ways in terms of economy as a whole. Similarly, Barajas et al. (2009) uncovered the outcome of remittances on economic expansion for a panel of 84 economies spanning data from 1970 to 2004. They used the PLS and fixed effects instrumental variable method and revealed that remittances in maximum cases, produced a negative sign, while in other cases, no robust association was observed between remittances and growth. Moreover, they commented that though remittances elevated some people from poverty, did not push them to be entrepreneurs.

In the ‘remittance-growth nexus’, the above-cited studies considered a linear impact of remittance on the latter, none of them ever investigated the possible asymmetric effect of remittance on EG. Moreover, earlier studies examined the relation of EG with FD, INQ, and personal remittances separately. The present study combines all these variables into a single model. In short, the novelty of the current study lies in the fact that ① to the best of the author's understanding any study on the top ten remittance-receiving countries is yet to be reported. ② This study examines the asymmetric effect of remittance inflow on economic progress, which is also a new dimension to the remittance-growth relationship. ③ It also captures the influence of FD on remittance inflow, which is a new addition to the literature, because the previous studies particularly examined the impact of remittances on FD. ④ It considers the recently devised composite FD (FD) index by the IMF, while the previous studies used different proxies such as domestic credit to the private sector, deposit-GDP ratio, and broad money, narrow money–GDP ratio. ⑤ It incorporates INQ and FD as independent variables into the conventional remittance-growth relationship and examines their impact on the remittance-growth nexus by employing the PMG estimate and D-H causality check.

## 3 Methodology

We intend to examine the impact of remittance inflow, financial development, and institutional quality on economic growth. The macroeconomic behavior of remittance inflow is fluctuating in nature and very often follows a cyclical pattern. Several studies such as Imai et al. (2014) and Islam (2022) exhibited this phenomenon. Therefore, we adopt an asymmetric association between personal remittances and EG. There are several recent studies such as Samargandi et al. (2015), Peprah et al. (2019), and Matei (2020) that have considered the non-linear association between “FD and EG”. Following them, this study considers a non-linear relation between FD and EG. Moreover, Thus, the remittance-growth nexus is specified in Eq. (1), which predicts the asymmetric impact of remittances, non-linear impact of FD, and linear impact of INQ on the EG of the 10 largest remittance-receiving nations of the world. The source of data on GDP, personal remittances, and INQ is the World Bank, while that of FD is the International Monetary Fund (IMF).

(1) $$LnGDP=f\left(REM,FD,FD^{2},INQ\right)$$

The top ten countries are India, China, Mexico, Philippines, Egypt, Pakistan, France, Bangladesh, Germany, and Nigeria, which remained at the top of the list of remittance-receiving countries in the world in 2020 (World Bank, 2021). The variables and their descriptive statistics are outlined in Table 1.

**Table 1:** Variables’ descriptions.

Variable	Description	Mean	Max	Min	Std. dev	Obs.
LnGDP	Natural logarithm of GDP at 2010 constant US dollars.	27.16	30.08	24.74	1.40	240
REM	Personal remittance inflow as a percentage of GDP.	3.73	12.78	0.03	3.29	240
FD∗	Composite FD index constructed by the International Monetary Funds.	0.40	0.84	0.13	0.19	240
FDI	Foreign direct investment inflow is a fraction of GDP.	1.97	12.76	−0.73	1.50	240
INQ†	INQ index derived from the World Bank governance data.	-0.21	1.60	-1.26	0.85	240

Source: The World Bank (2021) and the IMF (2021).

∗ FD is a composite index of country's relative rank of its “financial market and institutions” on their “depth, access and efficiency”.

† The INQ (institutional quality) is prepared based on the “Worldwide Governance Indicators” which has six indicators on various dimensions of governance. They include, “voice and accountability, political stability and absence of violence/terrorism, government effectiveness, regulatory quality, rule of law, and control of corruption”. We have combined these indicators into a single indicator taking their average and defined the INQ index (Kaufmann et al., 2010).

The data span includes from 1996 to 2019, which is decided by the availability of data. A larger data span though exists for GDP, REM, and FD; the data on INQ is available from 1996 onward. We employ a panel of the above data. In any panel study, before conducting any estimation, it is necessary to determine whether the cross-sections are correlated to each other. If the cross-sections are independent as most studies assume, then the first-generation unit-root test is used, otherwise, a second-generation unit test must be employed. Therefore, this study employs Pesaran's (2004) cross-section dependency (CD) test to ensure the dependency of the cross-sections. Based on the CD test outcomes, available second-generation unit root tests (Pesaran, 2007; Pesaran et al., 2009) are carried out to confirm the stationarity properties of the cross-sections. If the cross-sections are stationary, then only we can proceed with the estimation process.

Based on the stationary properties, we apply Pesaran et al. (1999) PMG estimation, which is suitable for different integrating orders and to capture the asymmetric influence of dynamic regressors on the dependent variable. The nature of personal remittances is asymmetric and is repeatedly unstable and unpredictable owing to the economic environments of the host countries from where remittances are sent to the receiving countries. Hence, we examine the asymmetric impression of remittances on EG, and the positive and negative components (shocks) of remittances are found utilizing the partial sum process expressed in Eqs. (2) and (3).

(2) $$R{REM\_P}_{t}=\sum_{i=1}^{t}{\Delta REM\_P}_{t}=\sum_{i}^{t}\;\max\;\left({\Delta REM}_{i},0\right)$$

(3) $${REM\_N}_{t}=\sum_{i=1}^{t}{\Delta REM\_N}_{t}=\sum_{i}^{t}\min\;\left({\Delta REM}_{i},0\right)$$

The dynamic procedure of the PMG model is outlined in Eq. (4).

(4) $$LnGDP_{it}=\mu_{i}+\sum_{j=1}^{l}\vartheta_{ij}LnGDP_{i,t-j}+\sum_{j=0}^{m}\theta_{ij}X_{i,t-j}+\varepsilon_{it}$$

where, i = 1, 2,…,N, symbolizes the quantity of cross-sections, t = 1, 2, 3,…,T, denotes time, j represents the time lag, $X_{i}$ stands for the vector of explanatory variables, and $\mu_{1}$ stands for fixed effect. We re-parameterize Eq. (4) and re-specify it in the following form.

(5) $${\Delta LnGDP}_{it}=\mu_{i}+\beta_{i}{LnGDP}_{i,t-1}+{\gamma_{i}X}_{it}+\sum_{j=1}^{l-1}\delta_{ij}^{*}{\Delta LnGDP}_{i,t-j}+\sum_{j=0}^{m-1}\theta_{ij}^{*}\Delta X_{i,t-j}+\varepsilon_{it}$$

where $\beta_{i}=-1(-\sum_{j=1}^{l}\delta_{ij});\;\gamma_{i}=\sum_{j=0}^{m}\theta_{ij})\text{.}$

We group the variables further at levels and rewrite Eq. (6) in the error correction method.

(6) $${\Delta LnGDP}_{it}=\sum_{j=1}^{l-1}\delta_{ij}{\Delta LnGDP}_{it-j}+\sum_{j=0}^{m-1}\theta_{ij}{\Delta X}_{it-j}+\rho_{i}\;\left[{LnGDP}_{it-1}-{\gamma_{i}X}_{it-1}\right]+\mu_{i}+\varepsilon_{it}$$

$\rho_{i}$ stands for the error-correction (EC) coefficient, which measures the rapidity of correction of the dependent variable to its long-term stability resulting from possible deviation in explanatory variables [$X_{it}$]. If $\rho_{i}$ <0, a long-run relationship is ensured. Thus, a negative and significant value of $\rho_{i}$ confirms cointegration among the variables.

Lastly, we employ the Dumitrescu, and Hurlin (2012) causality check to identify the route of causation among the variables. This test assumes the coefficients to diverge across cross-sections; its model is delineated in Eq. (7).

(7) $$Y_{it}=\gamma_{i}+\sum_{i=1}^{k}\delta_{i}Y_{i,t-k}+\sum_{i=1}^{k}\theta_{i}X_{i,t-k}+\varepsilon_{i,t}$$

$\gamma_{i}$, $\delta_{i}$, & $\theta_{i}$ indicate a constant, lag parameter, and the coefficient's slope correspondingly. We specify the hypotheses, the null, and the alternative in the following way.

$$H_{0}:\delta_{i}=0,H_{1}:\left\{ \begin{array}{c} \delta_{i}=0,\forall_{i}=1,2,\ldots ..N\\ \delta_{i}\neq 0,\forall_{i}=N_{1}+1,N_{1}+2,\ldots ..N \end{array}\right.$$

The null hypothesis shows the presence of heterogeneous Granger causality for whole cross-sectional units, while the other one indicates the presence of at least one causal association in panel data.

## 4 Results

### 4.1 CD test results

We offer Pesaran's (2004) CD test outcomes in Table 2, which exhibit dependency among the cross-sections based on the CD values of the variables and their corresponding probabilities.

**Table 2:** Results of the CD test.

Variable	Value	p-value	corr	abs (corr)
LnGDP	32.23	0.000∗∗∗	0.981	0.981
REM	8.40	0.000∗∗∗	0.255	0.392
FD	5.87	0.000∗∗∗	0.179	0.402
FDI	2.88	0.004∗∗∗	0.088	0.252
INQ	6.15	0.000∗∗∗	0.187	0.402

CD ∼ N (0,1), ∗∗∗ shows significance at 1% level.

### 4.2 Panel unit test outcomes

As the cross-sections are interdependent, we conduct the second-generation stationary tests, and their outcomes are provided in Table 3. Based on the test outcomes, it appears that the cross-sections are stationary and their integrating order is different.

**Table 3:** Panel stationary test outcomes.

Variable	CADF test		CIPS test	
	Level	1st diff	Level	1st diff
LnGDP	−1.948	−1.921	−1.395	−2.215∗
REM	−2.406∗∗		−2.031	−4.436∗∗∗
FD	−2.156	−4.313∗∗∗	−2.516∗∗	
FDI	−1.854	−3.993∗∗∗	−2.495∗∗	
INQ	−1.878	−3.503∗∗∗	−1.732	−4.156∗∗∗

∗∗∗, ∗∗ & ∗ show significance at 1%, 5% and 10% levels respectively.

### 4.3 PMG model estimation outcomes

In the case of different integrating orders of the variables/cross-sections, the panel ARDL/PMG model is specifically suitable to apply. The lag order of the PMG model was obtained following a maximum of 2 lags for the dependent variable (LnGDP), while 1 lag for dynamic regressors [REM_N, REM_P, FD, INQ] based on the Akaike info criterion. The outcomes of the PMG model: ARDL (2, 1, 1, 1, 1, 1) are documented in Table 4.

**Table 4:** ARDL (2, 1, 1, 1, 1, 1) model estimation outcomes.

Variable	Coefficient	Std. error	t-statistic	Prob.
Long run equation				
REM_N	0.0273	0.0029	9.4584	0.0000∗∗∗
REM_P	−0.0127	0.0039	−3.2198	0.0016∗∗∗
FD	0.5695	0.2773	2.0542	0.0419∗∗
FDˆ2	−0.1489	0.2437	−0.6110	0.5422
INQ	0.0465	0.0191	2.4385	0.0160∗∗
Short-run equation				
ECT	−0.3436	0.112464	−3.0551	0.0027∗∗∗
Δ (LnGDP (−1))	0.3686	0.085142	4.3288	0.0000∗∗∗
Δ (REM_N)	0.0016	0.007904	0.1972	0.8439
Δ (REM_P)	−0.0666	0.054720	−1.2168	0.2258
Δ (FD)	−0.9813	0.672036	−1.4601	0.1466
Δ (FDˆ2)	0.9013	0.877867	1.0267	0.3064
Δ (INQ)	0.0049	0.035742	0.1360	0.8920
C	9.2417	3.037431	3.0426	0.0028∗∗∗
@TREND	0.013381	0.005330	2.510359	0.0132∗∗

∗∗∗ and ∗∗ show significance at 1% and 5% levels respectively.

The coefficient of REM_N is positive and significant at a 1% level, showing that the negative components of the personal remittances exert a positive impact on output causing a stimulus for economic progress. The coefficient of REM_P is negative and significant at a 1% level, showing that the positive components of the personal remittances exercise a negative effect on output causing a harmful influence on EG. However, the resultant overall consequence of the negative and positive shocks of personal remittances on EG is likely to be positive [(0.0273–0.0127) = 0.0146].

Moreover, it appears that there exists an obvious asymmetry between the effects of the positive and negative components of the remittance inflows. To validate this asymmetric influence, we conduct the long-run asymmetric test [C (1) = C (2)] and its outcomes are offered in Table 5. The asymmetric test outcome also validates the asymmetric relationship between personal remittance and economic expansion as the values of test statistics are significant at a 1% level.

**Table 5:** Long-term asymmetric experiment outcomes.

Test statistic	Value	df	Prob.
t-test	8.603906	135	0.0000∗∗∗
F-test	74.02720	(1, 135)	0.0000∗∗∗
Chi-square	74.02720	1	0.0000∗∗∗
H0: C (1) = C (2), Ha: C (1) ≠ C (2)			

∗∗∗ Indicates significance at a 1% level.

Hence, any increase in personal remittance inflow invariably influences the economic expansion of the top-ten remittance-receiving economies positively. This finding reinforces previous studies such as Islam (2021), Jushi et al. (2021), Chuc et al. (2021), Ekanayake and Moslares (2020), Adnan et al. (2020), Kruah (2017), Chowdhury (2016), Azam (2015), Imai et al. (2014), Islam and Imran (2013), Islam et al. (2012), Pradhan et al. (2008), Cooray (2012), Fayissa and Nsiah (2010), and Giuliano and Ruiz-Arranz (2009), which exhibited a positive influence of remittances on economic development. However, it opposes Chami et al. (2003) and Barajas et al. (2009) who reported negative impacts of remittances on growth. The outcomes have policy implications for governments to strive for more remittances and augment EG. They may enhance the migration of domestic surplus laborers, and ensure migration-friendly organizations by providing support facilities at various levels, which may attract further remittances and augment economic expansion.

FD generates a long-run positive coefficient, which is significant at 5% level. It elucidates that the impact of FD is positive on EG. Any improvement in FD is expected to augment the EG of the remittance-receiving countries. Moreover, the coefficient of $FD^{2}$ is though negative but highly insignificant, which fails to justify the threshold impact of FD on economic expansion. Hence, the relationship between finance and growth follows a linear form in the case of selected countries. The findings support the view of Arestis et al. (2001), Estrada et al. (2010), Masoud and Hardaker (2012), Qamruzzaman and Jianguo (2017), Misati et al. (2019), Olayungbo and Quadri (2019), Rahman et al. (2020) and Abeka et al. (2021), who publicized a positive and linear relationship between FD and growth, while contradicts with Samargandi et al. (2015), Peprah et al. (2019) and Matei (2020) who highlighted an inverted U-shaped association between finance and growth. The outcome has policy implications for the policymakers to improve the “depth, access, and efficiency” of financial deliverables and further advance their “financial institutions and financial markets” to promote FD and stimulate EG.

The INQ also produces an affirmative coefficient, which is significant at 5% level. It demonstrates that INQ has an impressive influence on economic development. Any improvement in the INQ is expected to expand the EG performance of the economies. The outcome is in line with Zhao et al. (2021), Marija (2020), Alexiou et al. (2014), Abubakar (2020), Nawaz et al. (2014), Salman et al. (2019), and Nguyen et al. (2018), who conveyed vital influence of INQ on economic progress. This finding has policy implications for the concerned countries to enhance their INQ which has broad dimensions to improve. For example, increasing the voice and accountability, political constancy, the effectiveness of the government, regulatory quality, and rule of law; while reducing violence/terrorism and corruption is necessary to improve INQ.

The short-term estimates show that the coefficient of Δ(LnGDP (−1)) is affirmative and significant at the 1% level that justifies the autoregressive nature of the model. The lag values of GDP cause its growth. None of the variables show any significant short-term impact on EG. The coefficient of ECT is negative [−0.3436] having a 1% probability, which justifies the long-term correlation among the variables and exposes the rapidity of correction to the long-term stability from any short-term disequilibrium. It suffices that the model will always arrive at a long-term stable position at the rapidity of 34.36% per year from any short-term imbalance.

### 4.4 Robustness check with an additional variable

An additional variable, namely, FDI (foreign direct investment) inflow is added to the PMG model estimation to check the robustness of estimation outcomes. We add FDI as an extra variable into the existing model, because it is a similar source of external finance widely studied in literature to examine its impact on EG. The estimated output of the augmented model [with FDI] is reported in Table 6.

**Table 6:** ARDL (2, 1, 1, 1, 1, 1) output augmented with FDI.

Variable	Coefficient	Std. error	t-statistic	Prob.
Long run equation				
REM_N	0.024884	0.003264	7.623566	0.0000∗∗∗
REM_P	−0.009595	0.003565	−2.691589	0.0080∗∗∗
FD	0.414919	0.095029	4.366254	0.0000∗∗∗
FDI	0.004250	0.001371	3.100277	0.0024∗∗∗
INQ	0.041216	0.021382	1.927608	0.0560∗
Short-run equation				
ECT	−0.345315	0.113884	−3.032176	0.0029∗∗∗
Δ (LnGDP (−1))	0.358516	0.080058	4.478190	0.0000∗∗∗
Δ (REM_N)	0.001487	0.005689	0.261296	0.7943
Δ (REM_P)	−0.079381	0.052799	−1.503455	0.1351
Δ (FD)	−0.012672	0.077298	−0.163932	0.8700
Δ (FDI)	−0.000378	0.002310	−0.163626	0.8703
Δ (INQ)	0.019885	0.035629	0.558110	0.5777
C	9.271258	3.060492	3.029336	0.0029∗∗∗
@TREND	0.013183	0.005311	2.482335	0.0143∗∗

Note: ∗∗∗, ∗∗ and ∗ show significance at 1%, 5% and 10% levels correspondingly.

The estimated outcome of the augmented model shows similar long-run impacts of all variables including a positive impact of FDI on EG. The speed of error correction of 34.53% also remains very close to that of the initial model, 34.36%. Hence, augmented model outcomes authenticate the robustness of the estimations.

### 4.5 Adding interaction term, FD∗REM

The model is further estimated by adding an interaction [FD∗REM] variable FDRM to see the interacting effects of financial development along with remittance inflows. The outcomes are portrayed in Table 7.

**Table 7:** ARDL (2, 1, 1, 1, 1) output with interaction term.

Variable	Coefficient	Std. error	t-statistic	Prob.
Long run equation				
REM	0.0021	0.0062	0.3377	0.7361
FD	0.3332	0.0523	6.3704	0.0000∗∗∗
INQ	0.0365	0.0271	1.3487	0.1794
FDRM	0.0152	0.0177	0.8564	0.3931
Short run equation				
ECT	−0.3794	0.1096	−3.4621	0.0007∗∗∗
Δ (LnGDP (−1))	0.3453	0.0838	4.1199	0.0001∗∗∗
Δ (REM)	−0.1633	0.1806	−0.9042	0.3673
Δ (FD)	−0.0622	0.2130	−0.2919	0.7707
Δ (INQ)	0.0018	0.0343	0.0522	0.9585
Δ (FDRM)	0.1893	0.2264	0.8360	0.4044
C	10.3608	3.0841	3.3594	0.0010∗∗∗
@TREND	0.0104	0.0025	4.1977	0.0000∗∗∗

Note: ∗∗∗ shows significance at 1% level.

The estimated outcomes of the model with the interaction variable are somewhat dissimilar from those of the asymmetric model. In contrast with the asymmetric model, the long-run linear impact of remittance inflows on LnGDP though positive turns down to be insignificant. This perhaps signifies the suitability of an asymmetric relationship between remittance inflows and EG in the economies under consideration, produced by the asymmetric model above.

The impact of FD remains positive as before. The impact of INQ on EG remains affirmative but turns out to be insignificant. Thus, the inclusion of the interaction term alters the significant coefficient into an insignificant one. Moreover, the interaction term FDRM itself provides a positive and insignificant coefficient, meaning that the interaction term does not influence the EG growth significantly in the selected countries. The outcome contradicts with Peprah et al. (2019) and Olayungbo and Quadri (2019) who highlighted the positive and negative impacts of the interaction term on EG respectively. Thus, the finding provides inconclusive evidence to determine whether the remittance-finance interaction is working in the proper direction. Nonetheless, it is safer to emphasize that financial development and remittance inflow are complementary to each other. The short-run coefficients are insignificant and the speed of error correction improves to 37.94% with an interaction variable.

### 4.6 D-H causality test outcomes

We employ the D-H causality check, with 1 lag order, and deploy the outcomes in Table 8. Five bidirectional and equal numbers of unidirectional causalities are detected among the variables.

**Table 8:** D-H causality test outcomes, lag 1.

Null hypothesis	W-stat.	Zbar-stat.	Prob	Decision
REM_P does not cause LnGDP	5.1530	7.370	0.000∗∗∗	Bidirectional
LnGDP does not cause REM_P	2.5221	2.5651	0.0103∗∗	REM_P ↔ LnGDP
REM_N does not cause LnGDP	1.2396	0.2227	0.8238	Unidirectional
LnGDP does not cause REM_N	7.16263	11.0404	0.0000∗∗∗	LnGDP→ REM_N
INQ does not cause LnGDP	3.0267	3.5376	0.0004∗∗∗	Bidirectional
LnGDP does not cause INQ	3.4155	4.2556	0.0000∗∗∗	INQ ↔ LnGDP
FD does not cause LnGDP	1.5745	0.8557	0.3922	Unidirectional
LnGDP does not cause FD	5.1424	7.4448	0.000∗∗∗	LnGDP → FD
INQ does not cause FD	2.7750	3.07288	0.0021∗∗∗	Bidirectional
FD does not cause INQ	3.4718	4.35970	0.0000∗∗∗	FD ↔ INQ
FD does not cause REM_N	4.4022	5.9989	0.0000∗∗∗	Bidirectional
REM_N does not cause FD	3.0274	3.4879	0.0005∗∗∗	FD ↔ REM_N
INQ does not cause REM_P	2.0989	1.7922	0.0731∗	Bidirectional
REM_P does not cause INQ	4.5145	6.2039	0.0000∗∗∗	REM_P ↔ INQ
FD does not cause REM_P	1.8236	1.28936	0.1973	Unidirectional
REM_P does not cause FD	4.8987	6.9056	0.0000∗∗∗	REM_P → FD
INQ does not cause REM_N	0.6926	-0.7764	0.4375	Unidirectional
REM_N does not cause INQ	2.0534	1.7090	0.0874∗	REM_N → INQ
REM_N does not cause REM_P	1.5019	0.7018	0.4828	Unidirectional
REM_P does not cause REM_N	9.8360	15.923	0.0000∗∗∗	REM_P → REM_N

∗∗∗, ∗∗ and ∗ show significance at 1%, 5% and 10% levels respectively.

The first feedback association exists between positive components of remittances and EG [REM_P ↔ LnGDP]. The first one-way causations exist from income growth to negative components of remittance [LnGDP → REM_N]. The positive shocks in remittances cause economic expansion and vice-versa. While economic growth also spurs negative shocks in remittance inflows. Thus, it appears that a rise in remittance inflows is likely to cause economic progress in the remittance-receiving nations, and hence, it suggests that policymakers and governments strive to attract more remittances to gear up economic expansion.

The second feedback relationship prevails between INQ and output growth [INQ ↔ LnGDP]. The second one-way causations exist from output growth to FD [LnGDP →FD]. These four causalities prove the robustness of the PMG outcomes.

The third bidirectional causality happens between FD and INQ [FD ↔ INQ]; FD enhances the INQ and vice-versa. Since, financial development promotes INQ, which in turn stimulates EG. Thus, FD causes economic expansion through the INQ channel as well.

The fourth bidirectional causation occurs between FD and negative shocks of remittance [FD ↔ REM_N], indicating a feedback relationship between them. The existing literature evidence that remittances very often cause FD; however, this study reveals reverse causation as well that FD also causes remittance inflows. This finding points out some specific indications for the policymakers to pay attention to FD to attract more remittance inflows through formal channels.

The last and fifth feedback relationship prevails between positive shocks of remittance and INQ [REM_P ↔ INQ]. The positive shocks of remittances cause INQ and vice-versa. Moreover, both of them have a direct feedback relationship with income growth exhibited by the first and second feedback relationships.

The third one-way causality follows from positive shocks of remittance to FD [REM_P → FD], indicating that the positive components of remittance inflow also cause FD. Thus, along with the fourth feedback relationship, it reveals that both the positive and negative shocks of remittances cause FD. This finding contradicts Olayungbo and Quadri (2019) who found no causality between remittances and FD and is in line with Mistasi et al. (2019) who reported the contributory impact of remittances on FD.

The fourth unidirectional causation takes place from negative shocks of remittances to INQ [REM_N→INQ], and the last & fifth one-way causality arises from the positive components of remittances to the negative components of the same [REM_P → REM_N].

## 5 Conclusion

The study has endeavored to examine the impact of FD and the quality of institutions on the remittances-EG nexus for the top ten remittance-receiving countries based on the PMG model estimation and D-H causality test. It has particularly analyzed the asymmetric influence of personal remittances on EG, as remittances often show asymmetric behaviors. The PMG estimation has confirmed the asymmetric influence of remittances on the EG of the countries under study. The positive shocks of the remittances influence EG negatively, while the negative shocks cause the latter positively. However, the overall impact of personal remittances on EG is encouraging, which follows the findings of most earlier studies in the literature.

Financial development also reinforces the positive influence on EG, and no evidence is found in favor of its threshold impact on EG. Thus, the finance-growth relationship follows a linear form as suggested by most of the studies in the literature. INQ also has shown a positive contribution to EG. Moreover, the augmented model with FDI inflow also demonstrates long-run similar impacts of explanatory variables on EG including a positive contribution of FDI to EG. The interaction term of FD and remittances also produces a positive coefficient indicating its contributory impact on EG. Thus, any improvement in FD and the quality of institutions is likely to add to the EG of the countries under consideration.

The D-H causality test produces five bidirectional and an equal number of unidirectional causations. They support the findings of thePMG estimations. FD stimulates INQ, which sequentially promotes EG. Thus, FD causes EG through the INQ channel as well. Both the positive and negative shockwaves of remittances cause FD, while FD causes negative shocks of remittances. It gives new evidence that FD also causes remittance inflows which is a new addition to the literature. Moreover, they also cause INQ, which in turn causes EG. Thus, remittance inflow, FD, and INQ directly or indirectly cause economic expansions in the top ten remittance-receiving countries.

The outcomes suggest implications for the governments and policymakers to carry on developing their “financial institutions and financial markets” to enhance the “depth, access and efficiency” of financial services, improve their INQ and strive for more remittances and augment EG.

### 5.1 Limitation

The study has examined the impact of FD and institutional quality on the remittances-growth nexus in the top ten remittance-receiving countries, using the PMG model. Further studies may comprise a larger sample and employ more advanced techniques.

## Declarations

### Author contribution statement

Md. Saiful Islam; Ibrahim A Alhamad: Conceived and designed the experiments; Performed the experiments; Analyzed and interpreted the data; Contributed reagents, materials, analysis tools or data; Wrote the paper.

### Funding statement

This research did not receive any specific grant from funding agencies in the public, commercial, or not-for-profit sectors.

### Data availability statement

Data associated with this study has been deposited at https://databank.worldbank.org/source/world-development-indicators#, https://data.imf.org/?sk=f8032e80-b36c-43b1-ac26-493c5b1cd33b.

### Declaration of interest's statement

The authors declare no conflict of interest.

### Additional information

No additional information is available for this paper.
